# An analysis of pre-service family planning teaching in clinical and nursing education in Tanzania

**DOI:** 10.1186/1472-6920-14-142

**Published:** 2014-07-12

**Authors:** Projestine S Muganyizi, Joyce Ishengoma, Joseph Kanama, Nassoro Kikumbih, Feddy Mwanga, Richard Killian, Erin McGinn

**Affiliations:** 1 Department of Obstetrics & Gynecology, Muhimbili University of Health Sciences (MUHAS), P.O. Box 65117, Dar es Salaam, Tanzania; 2 Engender Health, ACQUIRE Tanzania Project (ATP), P.O. Box 78176, Dar es Salaam, Tanzania; 3 Engender Health 440 Ninth Avenue, New York, NY 100001, USA

**Keywords:** Family planning, Teaching, Pre-service, Tanzania

## Abstract

**Background:**

Promoting family planning (FP) is a key strategy for health, economic and population growth. Sub-Saharan Africa, with one of the lowest contraceptive prevalence and highest fertility rates globally, contributes half of the global maternal deaths. Improving the quality of FP services, including enhancing pre-service FP teaching, has the potential to improve contraceptive prevalence. In efforts to improve the quality of FP services in Tanzania, including provider skills, this study sought to identify gaps in pre-service FP teaching and suggest opportunities for strengthening the training.

**Methods:**

Data were collected from all medical schools and a representative sample of pre-service nursing, Assistant Medical Officer (AMO), Clinical Officer (CO) and assistant CO schools in mainland Tanzania. Teachers responsible for FP teaching at the schools were interviewed using a semi-structured questionnaire. Observations on availability of teaching resources and other evidence of FP teaching and evaluation were documented. Relevant approved teaching documents were assessed for their suitability as competency-based FP teaching tools against predefined criteria. Quantitative data were analyzed using EPI Info 6 and qualitative data were manually analyzed using content analysis.

**Results:**

A total of 35 pre-service schools were evaluated for FP teaching including 30 technical education and five degree offering schools. Of the assessed 11 pre-service curricula, only one met the criteria for suitability of FP teaching. FP teaching was typically theoretical with only 22.9% of all the schools having systems in place to produce graduates who could skillfully provide FP methods. Across schools, the target skills were the same level of competence and skewed toward short acting methods of contraception. Only 23.3% (n = 7) of schools had skills laboratories, 76% (n = 22) were either physically connected or linked to FP clinics. None of the degree providing schools practiced FP at its own teaching hospital. Teachers were concerned with poor practical exposure and lack of teaching material.

**Conclusions:**

Pre-service FP teaching in Tanzania is theoretical, poorly guided, and skewed toward short acting methods; a majority of the schools are unable to produce competent FP service providers. Pre-service FP training should be strengthened with more focus on practical skills.

## Background

The link between family planning (FP) and population growth, health, and economic growth is well documented [1,2]. Thus, where the rate of FP use is low, human development indices are also low. Recent data indicate that sub-Saharan Africa (SSA) has the highest fertility rate, accounts for 50 percent of the world’s maternal deaths, and is the world’s most poverty-stricken region [3].

Despite the importance of FP in population growth, health, and economic growth, for many years FP teaching in pre-service medical and nursing education has remained inconsistent and widely varied in terms of extent and quality of acquired knowledge and skills [4-8]. In clinical practice, these training irregularities are manifested by the failure of programs to impart necessary practical skills to the students in order to enable them to provide quality and evidence based family planning services as graduates [5,9-11]. Although significant resources are often directed towards in-service training for FP, the advantages of investing in pre-service medical education far outweighs those of in-service training [11-13]. There is consistence among researchers on what should constitute pre-service curricula in order to be able to produce competent practitioners. Pre-service curricula should be competence or task-based giving more weight to a set of well defined, measurable procedural skills but without sacrificing theoretical knowledge [4,6,8,14]. In practice, however, inconsistencies in pre-service teaching in terms of quality and extent of competences and the means to evaluate them continue to be reported [6-8,14,15].

Currently published information on pre-service FP teaching in Sub-Saharan Africa is lacking. The scanty available information suggest that pre‒service FP teaching provide some counselling and FP skills but with a wide variation in the extent and quality of the training between and within countries [12]. Multiple problems in pre-service FP teaching have been raised including outdated curricula, reliance on theory, lack of teaching resources and national standards [12]. Tanzania is one of the countries in Sub-Saharan Africa where FP can be expected to make a big difference in improving the quality of life based on current FP statistics. Overall, the modern contraception prevalence rate among married women is 27 percent [16]. An additional 25% percent of currently married women have an unmet need for FP; this unmet need varies from 11 percent to 42 percent by region [16,17]. Demand for FP is high, with 44 percent of married women in Tanzania wanting to wait for at least two years before they become pregnant again, while 30% of women want no more children although it is only 4% who rely on permanent methods [17]. These statistics all point to an urgent need to expand access to quality FP services in the country.

Providers of FP services in Tanzania emanate from a wide range of pre-service training backgrounds. They include nurses at various National Technical Awards (NTA) levels and non-physician clinicians. Non-physician clinician cadres (such as assistant medical officers [AMOs] and clinical officers [COs]) are less-standardized internationally, but have been shown to play an important role in health service provision in developing countries including performing some of the major surgical procedures at the level expected of a physician in some settings [18,19]. Other FP providers include medical doctors and university graduate nurses.

According to Tanzania’s education system, teaching curricula for technical education courses (such as nursing and non-physician clinician training) are standardized according to the National Technical Awards (NTA) and accredited by the National Council for Technical Education (NACTE) [20]. NACTE standards stipulate that curricula should develop knowledge, skills, attitudes, and competencies in line with the expectations and needs of various stakeholders and future employers, such as the government/state, the academic world, the students and parents themselves, and society at large [20]. Likewise, higher level education such as Doctor of Medicine and nursing degree programs is accredited by the Tanzania Commission for Universities (TCU). According to TCU statement, higher education must respond to stakeholders expectations and ensure that it prepares young people to fit into their communities and lead productive lives [21]. The Inter-University Council for East Africa (IUCEA) is tasked with bringing together all the national committees in East Africa to harmonize teaching standards at the sub-regional level [22].

In view of the importance of FP to Tanzania, the broad educational background among FP providers and the paucity of information on pre-service curricula used to teach FP, there was need to explore the extent and quality of pre-service FP teaching. We formulated three primary questions: 1) Are the current curricula for pre-service training in clinical and nursing courses and programs suitable for FP teaching? 2) What proportion of pre-service courses and programs is in access to resources for FP skills training? 3) What level of competence should be expected of graduates of the various pre-service courses and programs in providing FP methods? The results of this study are expected to inform relevant institutions, policy makers and other stakeholders on areas for improvement and add to the current scanty literature on pre-service FP teaching in Sub-Saharan Africa.

## Methods

### Eligibility

All courses and programs (and implicitly their teaching institutions) providing pre-service clinical and nursing training and whose graduates are expected to provide FP services in Tanzania were eligible for inclusion in the study. Schools that offered these courses and programs were variably affiliated to institutions owned by the public, private, non-governmental and faith-based organizations. Pre-service technical education courses (i.e., certificate nursing, diploma nursing, clinical assistants (CA), clinical officers (CO), and assistant medical officers (AMO) were taught in 83 schools and together with six schools that teach degree programs made up a total of 89 schools eligible for the study. The degree programs included five Doctor of Medicine (MD) and one Bachelor of Science nursing (BSc Nursing). Ethical clearance was granted by the Muhimbili University of Health and Allied Sciences and the permission to conduct the study was obtained from the MOHSW and the teaching institutions. Verbal consent to participate in the study was requested and granted by all the participants.

### Sampling

We obtained updated lists of all the schools offering pre-service technical education courses and degree programs from the MOHSW and TCU respectively. These lists were used as our sampling frames. The distribution of the randomly sampled schools for courses and programs is summarized in Table 1. Overall 39 (44%) of all (n = 89) the eligible schools were sampled for evaluation. The sample includes all the schools teaching degree programs (n = 6) since universities are autonomous institutions. Likewise all AMO schools (n = 6) were included due to lack of a standardized curriculum for AMO course at the time of study. All other schools that provide technical education courses were stratified by their zonal locations according to the eight MOHSW zones in Tanzania. Schools in each zone were randomly sampled in order to include at least a third of schools for each course. Of the 39 representative schools, 35 (90%) were evaluated, 4 (10%) were not evaluated due to various reasons (Table 1).

**Table 1:** Distribution of schools according to study courses and programs

**Type of course/program**	**Total number of schools**	**Number sampled**	**Number evaluated**	**Reasons for not been evaluated**
Degree programs	6	6	5	Declined consent (n = 1)
Assistant Medical Officer (AMO)	6	6	4	On vacation (n = 2)
Clinical Officer (CO)	10	5	5	-
Clinical Assistant (CA)	7	4	4	-
Nurse (Diploma)	32	10	10	-
Nurse (Certificate)	28	8	7	On vacation (n = 1)
**Total**	**89**	**39**	**35**	**-**

### Data collection

We obtained teaching curricula for technical education courses from the MOHSW and degree curricula from the relevant degree institutions. In total, eleven curricula were obtained and evaluated for their suitability to guide pre-service FP teaching. These curricula include six NTA standard curricula and five degree program curricula (Table 2). One Doctor of Medicine (MD) curriculum was not analyzed due to denial of consent from the institutional authorities. There was one AMO syllabus which was prepared in 2000 by the MOHSW to guide AMO teaching, but not to NTA standards. This syllabus was also assessed. In addition to obtaining the curricula from MOHSW and degree institutions, we contacted key officials in the MOHSW, TCU, and NACTE for clarifications on various issues on curriculum development standards whenever the need arose. The documents and information from these sources were used in evaluation of the curricula for their suitability to teach FP.

**Table 2:** Distribution of the evaluated curricula by type of course/program

**Name of curriculum**	**Teaching course/program**	**Number evaluated**
Doctor of Medicine (MD)	MD	4
BSc. Nursing	Nurse (Degree)	1
Ordinary Diploma in Clinical Medicine (NTA LEVEL 6)*	AMO	1
Technician Certificate in Clinical Medicine (NTA LEVEL 5)	CO	1
Basic Technician Certificate in Clinical Medicine (NTA LEVEL 4)	CA	1
Ordinary Diploma Nursing (NTA LEVEL 6)	Nurse (Diploma)	1
Certificate Nursing (NTA LEVEL 5)	Nurse (Certificate)	1
Basic Certificate Nursing (NTA LEVEL 4)	Nurse (Basic)**	1
**Total**		**11**

*This curriculum was not in use yet at the time of study. In the meantime AMO training was partly guided by an old syllabus.

**This course was not taught in isolation at the time of this study but equivalent competences were included in the continuum of NTA curriculum.

Another source of information was the teaching institutions. Two senior doctors and a nurse who were experienced in medical, nursing teaching and research made visits to the institutions for data collection. All of them had participated in the piloting of data collection tools. They physically observed and documented teaching environment and assessed other available relevant FP teaching documents such as teaching time table, teaching notes, and practical evaluation books according to the checklist requirements. In addition they conducted semi structured interviews with one FP subject teacher from each of the visited teaching institutions. Some open questionnaires aiming at capturing interviewees’ perspectives on FP teaching were attached but the selection of which teacher to interview (n = 19) was done purposively among respondents of the main questionnaire. All the gathered information from this source was used to evaluate FP competence outputs of a school according to set criteria. Interviews with subject teachers were useful in determining gaps in availability of FP teaching resources at the institution. The information gathered from institutions was intended to be exploratory on FP teaching practice with regard to availability of resources, theory teaching and ability to provide temporary FP methods. Details on FP subject contents, step by step FP method provision process, and student attitude were beyond the scope of this study.

### Measures

We assessed the curricula from two perspectives; the quality of the curricula in terms of alignment with competence based format and the quality in terms of FP content in particular. Each curriculum was read thoroughly by two separate assessors who are trained in curriculum development and their consensus on each specified quality item was entered into a checklist for quantitative analysis. The criteria items used in our measure are in line with the recommendations for self-assessment of quality teaching/learning by the Inter-University Council for East Africa (IUCEA) and procedures for curriculum development, review, approval, and validation [22]. The items were discussed by the research team and NACTE/TCU experts.

#### Suitability of the curriculum for FP teaching

According to this measure the suitability of a curriculum for FP teaching was decided based on assessors’ agreement on the six item criteria. The curriculum was judged suitable to guide FP teaching if all of the six criteria items listed hereunder were fulfilled:

1. Inclusion of FP is not against the vision and mission of the institution.

2. FP topics are included in the curriculum.

3. The curriculum states expected FP competence outcomes of the graduate.

4. The curriculum shows explicitly how the expected FP competences will be measured.

5. The program demonstrates continuity in teaching of FP from less to more complex competences and arranged such that the knowledge and skills acquired at a lower level strengthens FP competence outputs at higher levels [i.e., it is expected students to acquire theory before practical skills; to observe demonstrations before self performing, to perform on models before clients and not the vice versa. Likewise skills taught at a lower level, say NTA-level 4 should not be more complicated than the skills acquired at NTA-Level 6 on the same subject].

6. All graduates of the program are exposed to FP teaching (i.e FP not optional).

#### The level of FP competence outputs of the school

Levels of FP competence output of a school for courses and programs were assessed based on the proof obtained about actual FP teaching, FP competence tasks, teaching resources and means of evaluation of FP competences. This measure was expected to rank FP competences expected of a graduate of a particular course or program from a particular school. In order to judge the expected level of FP competence outputs for any of the course, we verified the provision of both theoretical and practical training based on information collected from the institutions. Courses that were verified to offer FP theoretical training and whose graduates were trained via hands-on skill practice with real clients were judged likely to offer a high level of competence in FP service and all the rest were judged to produce graduates with low levels of FP competence. Criteria items for assessing the level of FP competence output of a course/program are listed below:

a) Students received theory training (Yes/No) AND

b) The level of practical (skills) developed/mastered by the graduate (listed in ascending levels of competence):

1. None (theory only)

2. Observing demonstrations only

3. Self performing on a model under supervision(depending on type of method) and

4. Hands-on skills including service provision to clients under supervision

#### Other documents

This assessment included additional material/documents we obtained from various sources. These include standard practical evaluation books for pre-service programs obtained from the MOHSW, procedure books from the institutions, and standard nursing procedures at the work place prepared by the MOHSW. The focus of assessment of these documents was to find additional supporting evidence regarding FP teaching and practice.

### Data analysis

Quantitative data thus generated from the above documents, observational checklists, and semi structured questionnaires were entered into a computer using EPI info 6 program cleaned and analyzed using standard methods. Responses to open questions were analyzed using Content Analysis whereby a sub-set of the comments was read and a coding frame generated to describe the thematic content of the comments. The codes were then assigned to all the comments and treated as variables in a quantitative analysis [23]. The design of the qualitative component, transparent interview procedures as afore described and this analysis conform to RATS guidelines (http://www.biomedcentral.com/authors/rats).

## Results

Among the assessed 35 schools, the presence of a copy of a curriculum in the school was verified in all of the five degree programs and 28 (93.3%) of all technical education courses. Technical education schools that did not possess a copy of NTA standard curricula include all 4 AMO and two nursing schools both of which affiliated to non-governmental organizations.

### The suitability of curricula for FP teaching

Seven curricula (63.6%) had FP included but only one among the assessed 11 curricula was judged suitable for FP teaching (Table 3). This suitable curriculum was the Technician Certificate in Clinical Medicine (NTA 5) for Clinical Officers. All the five degree program curricula had FP topics but none of them was qualified as suitable for FP teaching. In all of the degree curricula evaluation of FP competences was conspicuously lacking.

**Table 3:** Suitability of curricula for FP teaching

**Type of course/program**	**FP included**	**FP competence outcomes stated**	**Means of FP evaluation explicitly stated**	**Offers continuity in FP teaching**	**Suitable for FP teaching**
Doctor of Medicine (n = 4)	4 (100)	3 (75)	0 (0)	2 (50)	0 (0)
BSc. Nursing (n = 1)	1 (100)	1 (100)	0 (0)	0 (0)	0 (0)
NTA Clinical Medicine (n = 3)*	1 (33.3)	1 (33.3)	1 (33.3)	1 (33.3)	1 (33.3)
NTA Nursing (n = 3)**	1 (33.3)	0 (0)	0 (0)	0 (0)	0 (0)
**Total**	**7 (63.6)**	**4 (45.5)**	**1 (9.0)**	**3 (27.2)**	**1 (9.0)**

*Includes curricula for all clinical medicine courses (CA, CO, AMO) under NTA-level 4 to NTA-level 6 curricula for clinical Medicine.

**Includes curricula for all nursing courses (Basic, Certificate, Diploma) under NTA-level 4 to NTA-level 6 curricula for nursing.

AMO was found at only one of the four institutions visited. The syllabus had FP topics listed, but it did not meet standards for competence based curriculum development.

### Actual FP Teaching

It was verified that some theory FP teaching was offered by all the technical courses and degree programs. Diverse categories of FP methods were taught, including natural, short-acting, long-acting, and permanent methods. Long-acting methods were taught in all of the schools, followed by natural methods and short-acting methods (97% each) and permanent methods (93%). Likewise all the methods were theoretically covered in all the degree programs. In all the 35 courses and programs, the documented duration of theory teaching ranged from 2 to 75 hours.

Some form of practical FP teaching was claimed by twenty eight (93%) of technical education schools and all of the degree schools. Nevertheless none of the assessed practical modules or sessions in all these schools was devoted for FP. These modules or sessions were rather a community health field rotation or a Reproductive health (RCH) rotation, which made it difficult to estimate exposure time for FP practical activities per se. According to observations and assessment of various institutional documents frequencies of FP method demonstration were: Oral contraceptive pills (97%), injectables (83%), IUCDs (77%), implants (77%), barriers (73%), surgical methods (53%) and spermicides (50%).

Table 4 shows the availability of practical teaching facilities for technical education courses. Only 23.3% (n = 7) of schools had skills laboratories, 76% (n = 22) had FP clinics to train the students, 70% (n = 21) were affiliated to hospitals where they could observe surgical methods.

**Table 4:** Technical education schools in access to FP teaching resources, N = 30

**Type of FP teaching resource**	**Schools in access**	
	**n%**	
Skills laboratory	7	23.3
FP clinic	22	73.3
Hospital for FP practice	21	70
Maternal and child Health (MCH) services	27	90

None of the degree program had a skills laboratory for FP and none utilized its university affiliated hospital or FP clinics for FP practice.

### Evaluation of FP competences

Evaluation of FP competences was also lacking. In 13.3% (n = 4) of the technical education courses there was either no evaluation of practical FP at all (n = 3), or no specific evaluation tool used. Thirteen (43.3%) of the technical education schools relied on standard tools produced by the MOHSW and a similar percentage relied on their locally designed evaluation tools. For BSc Nursing School, FP practice was conducted in the community where the students were expected to practice some FP hands-on skills. However, there were no specified competence tasks and a tool to evaluate FP skills. Likewise, all schools that provide Doctor of Medicine (MD) program assumed that the students could observe FP methods during field training in the community but none of these programs had specified competence tasks or tool to evaluate FP skills.

### FP competences outputs

Overall only 22.9% of the evaluated schools for pre-service courses and programs met the criteria for high competence level ranking (Table 5). These graduates were considered able to skilfully provide short- and long-acting methods to clients immediately after graduating. No degree program was better than technical education courses in this regard. Only one school (for AMO) produced graduates who could provide surgical methods of contraception.

**Table 5:** Distribution of schools, courses and programs whose competence outputs were ranked high level

**Type of course/program**	**Total evaluated schools**	**Ranked high level competence**	
		**n%**	
MD	4	0	0
BSc. Nurse	1	0	0
AMO	4	1	25
CO	5	2	40
CA	4	0	0
Nurse (Diploma)	10	4	40
Nurse (Certificate)	7	1	14.3
**Total**	**35**	**8**	**22.9**

### Comments by informants

Nineteen FP instructors from 19 institutions were purposively selected and willing to comment on FP teaching at their working place. The comments were summarized into 7 thematic categories. The categories are listed below with the number of informants in brackets:

1. Concerns on the lack or inadequate FP practical exposure for students (n = 9)

2. Lack of teaching resources (including teaching equipment, facilities, FP methods/updated teaching material) (n = 6)

3. Concerns with curriculum inefficiencies (n = 5, all from technical education courses)

4. Lack of FP teachers (n = 4, all from technical education courses)

5. Irregularities in evaluation, including examinations(n = 4)

6. Intimidation of FP teachers by faith authorities (n = 3, all from FBO owned or associated courses)

7. Need for knowledge and skills update for FP teachers (n = 2)

## Discussion

Our main tasks in this study were to assess the current pre-service curricula and the actual teaching on FP at a selection of pre-service medical/technical schools in Tanzania, in order to identify any gaps and suggest opportunities for strengthening the teaching. In this regard, we assessed 11 recognized pre-service curricula, one syllabus and a total of 35 representative schools that offer pre-service teaching of FP in Tanzania.

We identified four curricula without FP topics. These curricula were for instruction of basic nurse, diploma nurse, clinical assistant and assistant medical officer courses whose graduates are expected to assist health workers in the provision of preventive, curative, diagnostic, and administrative services in health care settings with application of their competence at routine levels [20,22]. Although a clear definition of ‘routine level’ was not included in the National Technical Awards curricula, there is evidence that FP knowledge and skills are widely-applicable in many day-to-day reproductive and maternal health services, such as maternal and child health (MCH) services, as well as in prevention of sexually-transmitted infections, including HIV. The availability of FP at all service levels is therefore considered a basic health requirement [11,24].

Clinical assistants and nurses trained to basic certificate nurse level (NTA levels 4) are expected to work mainly in frontline health facilities where a variety of FP methods is generally lacking [25]. If they are not taught on FP while in school these cadres will not have sufficient knowledge and skill to be able to assist other health workers in providing FP methods in the frontline health facilities. Furthermore, after graduation, these providers are unlikely to acquire or improve their knowledge and skills on FP due to a limited exposure to a variety of FP methods and in-service training programs.

The exclusion of FP teaching in ordinary diploma (NTA level 6) curricula for both nursing and clinical medicine courses apparently assumed that, FP should have been taught during a certificate course for nursing and clinical medicine (i.e., during NTA level 5) since NTA curricula assume a step by step progress from the lowest level to the highest. This assumption, however, can only be reliable if the programs strictly assumed a stairway system of moving from NTA level 4 to 6. In practice, however, it is possible for the graduate at NTA level 4 to bypass NTA-level 5 and join NTA level 6 directly as an in-service entrant. If this happens, there is a possibility of such NTA 6 graduates to completely lack the necessary knowledge and skills in FP practice.

For those curricula that addressed FP, a number of irregularities were noted that indicate they were largely inadequate in content and approach. In some cases, modules with some form of practical FP training preceded the semester during which FP theory module was supposed to be introduced, and in other curricula, there was no reconciliation between the contents of the course modules and the expected competencies. Such irregularities have been mentioned by NACTE and TCU as contrary to effective teaching and learning, and did not conform with standard guidelines for a quality program [20,21].

Overall, the inclusion of FP in the assessed curricula was not as prominent as one would expect, given the importance of FP as a common health intervention and its relevance to the country’s development policies. Of all the analysed curricula, no curriculum had FP as an independent module. In most curricula, FP was a short topic in a module—a sub-topic or just listed in few bullets. In all cases, FP was included in the same module with other reproductive and child health (RCH) services or midwifery topics. In these curricula, other aspects of RCH or midwifery were almost always given more weight than FP. For example, in one nursing program, the number of deliveries conducted was set as a criterion for a successful exit at NTA-level 6, while there was no mention of the skills required for FP, even though both were included in the same module.

In most degree programs there were no specific FP competences mentioned in the specific objectives for a semester or module, hence the means of evaluation were generally less-suitable for FP skills. Most evaluations in the degree programs relied on traditional methods, such as seminar with or without case presentation and observations, both of which have limitations for practical skills teaching. For instance, seminars do not present a real-life situation for the students to perform clinical skills [14,26]. Observing the procedure being done without self-performing is unlikely to impart skills and confidence to the level required for quality service provision. Isolated examinations for students on FP skills, done at the end of a semester, are not likely to improve students’ skills. This approach lacks an opportunity for feedback and a chance to exercise those skills once the student has advanced to the next semester.

Theory was the mainstay of pre-service FP teaching. Most technical education schools and degree schools had no facilities or arrangements to facilitate effective practical FP teaching. Furthermore, teachers for many technical education courses were not updated on FP issues and some of the schools relied on part-time teachers. Across pre-service FP teaching programs with FP competencies implied or specified, the targets were similar regardless of the level of the educational program. For instance, the expectations of a certificate nurse were similar with those of a medical doctor, BSc nurse, AMO and a clinical officer. This partly reflected the lack of a national strategy for FP teaching and the failure to adapt and implement a competency-based national qualification system for FP, which has been instrumental in improving pre-service FP teaching in other countries [11].

Family planning topics in all courses and programs were skewed toward short acting methods of FP. None of the analysed programs targeted surgical methods of contraception, and, perhaps contributing to only 9 percent of health facilities in Tanzania offering surgical methods of contraception in contrast to more than 70 percent of the facilities that offer short acting methods [25]. This skewed trend toward non-surgical methods of contraception has been attributed mainly to the lack of skills of the teaching staff [25]. This gap is therefore not adequately addressed by the current technical and medical education systems, as supported by the comments from the interviewed participants.

Our analyses have indicated that only nearly a quarter of all the pre-service schools were judged to have high FP competence outputs. This can be interpreted that only these few schools have resources in place to produce competent graduates able to perform skilled provision of FP methods. Moreover, students in pre-service schools were not equally exposed to all FP methods indicating that some graduates may not even know some of the FP methods. A similar pattern was reported in other medical education systems elsewhere [9-11,27], calling for urgent review of current teaching curricula.

Among the potential limitations of this study is the non-inclusion of provider attitudes towards FP in our evaluation, the failure to assess FP teaching in four schools and the failure to interview students. Moreover, we did not analyze to what extent teaching materials were evidence-based, although the comments made by teachers made it apparent that there was lack of updated teaching material. As both provider attitudes and updated teaching material are important determinants of competence development [11], future studies should include an examination of these elements in pre-service FP teaching. This study was just exploratory and not meant to exhaustively assess every item in FP teaching. Our results have sufficiently answered our objectives and shone light on areas for improvement and further research.

Finally this study was conducted at the time when NTA curricula were undergoing minor necessary changes. Likewise a review of the TCU standardized curricula was underway. In this respect, some of the suggestions made in this article with regards to NTA curricula may have already been implemented. However, the timing of this curricula review may be advantageous for relevant curriculum developers who may find our suggestions useful and worthy of consideration.

## Conclusions

In conclusion, while all pre-service medical programs have the potential to include FP teaching without contradicting the vision and missions of their institutions, only one teaching curricula was suitable for FP teaching. Schools lack skills training facilities; the problem is more acute for degree offering schools than non-degree ones. Overall, a third of all the pre-service schools could produce graduates competent enough to provide short acting and long acting FP methods. We recommended re-addressing FP training in Tanzanian pre-service education, based on its current importance to national needs and regional/international curriculum development standards.

## Competing interests

The authors declare that they have no competing interests.

## Authors’ contributions

PS contributed to the planning and design of the study and was involved in data collection, analysis and manuscript writing. JI was involved in planning, designing, data collection, analysis and manuscript writing. JK, NK, FM and RK were involved in planning, design and manuscript writing. EM was involved in planning, design and manuscript writing. All the authors read and approved the final draft of the manuscript.

## Pre-publication history

The pre-publication history for this paper can be accessed here:

http://www.biomedcentral.com/1472-6920/14/142/prepub
